# Dry textile electrode for ambulatory monitoring after catheter ablation of atrial fibrillation: A pilot study of simultaneous comparison to the Holter electrocardiogram

**DOI:** 10.12688/f1000research.75712.2

**Published:** 2022-06-30

**Authors:** Takeshi Machino, Kazutaka Aonuma, Yuki Komatsu, Hiro Yamasaki, Miyako Igarashi, Akihiko Nogami, Masaki Ieda

**Affiliations:** 1 Department of Cardiology, Faculty of Medicine, University of Tsukuba, Tsukuba, 305-8575, Japan; 2 Department of Clinical Research and Regional Innovation, Faculty of Medicine, University of Tsukuba, Tsukuba, 305-8575, Japan

**Keywords:** atrial fibrillation, textile electrode, wearable electrocardiogram, ambulatory monitoring, catheter ablation

## Abstract

**Background**: Holter electrocardiogram (ECG) is the gold standard for ambulatory monitoring of atrial fibrillation (AF) but it is insufficient because of its limited recording time. Although several consumer ECG devices provide longer recording time, they generally do not undergo the regulatory process for medical use. Furthermore, current medical-grade devices for longer ECG monitoring are not continuous or too invasive for AF monitoring. A wearable ECG with a medical-grade dry textile electrode is a promising technology to remedy this limitation. This pilot study aimed to simultaneously compare the wearable and Holter ECGs for ambulatory monitoring in a clinical setting.

**Methods: **This prospective observational study enrolled 18 patients who underwent AF ablation. One day after AF ablation, ambulatory ECG was obtained for three hours simultaneously using both the wearable and Holter ECG devices. Automatic ECG interpretations between devices were compared with correlation and agreement analyses.

**Results:** Simultaneous ECG monitoring demonstrated a comparable analysis time and total heart beats between the two devices. Almost complete correlation and agreement were also demonstrated in all clinically relevant testing aspects except in R-wave amplitude (r = 0.743, p < .001). AF was detected in three patients. AF duration was the same in both ECG devices in two patients with continuous AF. In the remaining patient with intermittent AF, AF duration was shortened by 0.6% with the wearable ECG as compared to that with the Holter ECG.

**Conclusions: **Simultaneous ECG comparison revealed a high consistency between the wearable and Holter ECG devices. The results of this study warrant further clinical studies for long-term monitoring of ambulatory ECG after AF ablation.

## Introduction

Atrial fibrillation (AF) is the most prevalent form of arrhythmia with an increasing incident worldwide; it is associated with an increased lifetime risk of stroke, heart failure, myocardial infarction, dementia, and mortality. ^1^^,^^2^ Whether AF presents or not is crucial for making a clinical decision regarding treatment strategy. Accordingly, long-term monitoring with an electrocardiogram (ECG) is essential for the management of AF, which can present with short and silent forms, especially those treated with catheter ablation. ^3^^,^^4^

Conventional ECG monitoring for medical examination is limited to 24 h. Although long-term monitoring devices have revealed under-detection of AF via Holter ECG, ^5^^,^^6^ they also have shortcomings. A cardiac event recorder cannot provide continuous monitoring, while an implanted loop recorder is too invasive for mere AF monitoring. Although several consumer devices have been marketed recently, their ECG results are sometimes not precise and are very noisy. ^7^ Furthermore, those devices generally do not undergo the regulatory process for medical use. Therefore, AF patients are still in need of a medical-grade device for long-term non-invasive and continuous ECG monitoring.

Recently, a novel dry textile electrode “hitoe ^®^” (Toray Industries Inc., Tokyo, Japan) has been registered as a medical device for wearable ECG in Japan (13B1X0001500034). The hitoe ^®^ is highly conductive for recording ECG in a non-invasive and continuous manner. ^8^ Wearable ECG with hitoe ^®^ demonstrated its usefulness for a few minutes in healthy volunteers. ^9^ However, consistency between the wearable and Holter ECG has not been evaluated simultaneously in clinical settings. Therefore, this pilot study aimed to simultaneously compare both devices for ambulatory ECG monitoring after AF ablation.

## Methods

### Recruitment

This prospective observational study enrolled 18 patients (14 males and 4 females) with AF who were admitted for catheter ablation to the University of Tsukuba Hospital from August 2017 to March 2018. Eligible patients were aged 20 years or older (legal adult in Japan) with an under-bust size between 84 and 100 cm (suitable circumference for the wearable ECG). To avoid the potential risk of skin-related issues associated with ECG monitoring, we excluded patients with known skin allergy and skin sensitivity to adhesive tape as denoted by a history of redness, erosion, and scarring post exposure.

All participants provided written informed consent before enrollment. This study complied with the Japanese Ethical Guidelines for Medical and Health Research Involving Human Subjects as well as the Declaration of Helsinki. The study protocol was approved by the Review Committee of University of Tsukuba Hospital (H29-88).

### Study design

**Figure 1.**
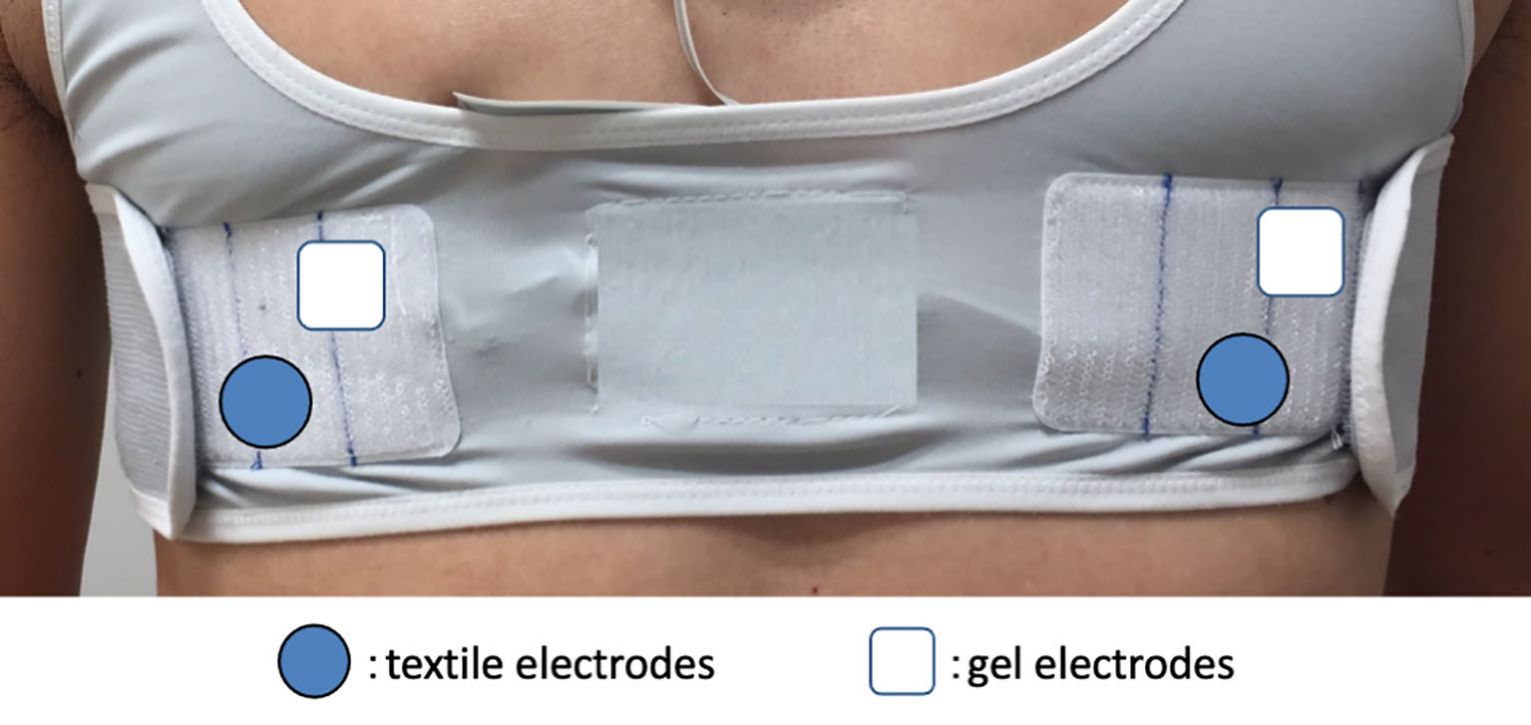
Simultaneous positioning of textile and gel electrodes. Bipolar electrodes for CC5 lead were positioned to avoid interference between textile and gel electrodes.

Ambulatory ECG was simultaneously obtained from both wearable and Holter ECGs for 3 h on the day after AF ablation, which mainly consisted of electrical isolation of the pulmonary veins from the left atrium. ^10^ The ECG was recorded via a bipolar CC5 lead from dry textile electrodes (wearable ECG) as well as via wet gel electrodes (Holter ECG). Both ECG recordings were interpreted by the same automatic analyzer to avoid a reporting bias. The simultaneous ECG comparison eliminated length and lead-time biases between the two devices. In addition, a dropout bias was reduced by an in-hospital ECG recording on the day after AF ablation. All electrodes were carefully positioned to avoid interference from each other ( Figure 1).

The wearable ECG system was developed by Toray Industries Inc. (Tokyo, Japan) as a smart bra-type device equipped with the dry textile electrode (hitoe ^®^). The electrode consisted of woven nanofibers coated with a highly conductive polymer (PEDOT-PSS). An insulated electro-conductive lead ribbon was connected between the electrodes, and a connector terminal was utilized for signal transmission. ^9^

Holter ECG recording was obtained using a conventional Ag/AgCl electrode (Cardyrode-P ^®^, SUZUKEN Co., Ltd., Nagoya, Japan). An electroconductive gel surrounded the electrode for skin adhesion. The conventional electrodes were equipped with connecting cables between them and utilized a connector terminal for signal transmission.

Because both devices were not waterproof, bathing and showering were prohibited during the three-hour study period. MRI was also prohibited to avoid thermal skin injury secondary to an induced electromotive force evoked by time-varying magnetic fields in the presence of electrical monitoring devices. ^11^

### Measurements

The ECG signal was transmitted to a Holter ECG recorder (Kenz Cardy 303 Pico+ ^®^, SUZUKEN Co., Ltd.). Recordings obtained from the wearable and Holter ECG devices were interpreted using the same automatic analyzer (Kenz Cardy Analyzer 05 ^®^, SUZUKEN Co., Ltd.).

Analysis time was defined as the signal recording time after artifact removal. Tiny spikes, notches, large baseline swings, widened isoelectric line, loss of ECG signal, and electromagnetic interference were manually removed as artifacts. ^9^ Based on template matching, the automatic analyzer categorized QRS morphologies into a normal QRS complex, atrial premature complex (APC), ventricular premature complex (VPC), and noise signal. Noise signals were excluded from the calculation of total QRS complexes.

The R-wave amplitude was measured from the PQ segment to the top of the R wave and was averaged over 10 consecutive beats of normal QRS complexes, which were obtained simultaneously from both devices for comparison. AF was detected based on any RR interval irregularity lasting over 30 s. ^12^ Noise signals and VPCs were excluded from the RR interval analysis for AF detection. The number of AF episodes and the total AF duration were compared between the wearable and Holter ECG.

### Statistical analysis

Categorical variables were summarized as numerical counts (percentages). Continuous variables were expressed as mean ± standard deviation (SD) or as median (interquartile range [IQR]) and were compared using the paired t-test or Wilcoxon signed-rank test. Normality was assessed using the Shapiro-Wilk test. Agreement of measurements between the wearable and Holter ECG was assessed with the Bland-Altman analysis for normally distributed variables. Alternatively, the Passing-Bablok analysis was used for non-normally distributed variables. Correlations between the wearable and Holter ECG were evaluated using Pearson’s or Spearman’s analysis. The minimum sample size to detect a correlation coefficient (≥ 0.70) differing from zero (power 0.8; alpha 0.05) was 13 for Pearson’s and 15 for Spearman’s analysis. ^13^ A two-tailed *P*-value < .05 was considered significant. Statistical analyses were performed using IBM SPSS Statistics for Macintosh, Version 26 (IBM Corp., Armonk, N.Y., USA) (RRID:SCR_019096) and SciStat Version 2.9 (MedCalc Software Ltd., Ostend, Belgium) (RRID:SCR_021918).

## Results

**Figure 2.**
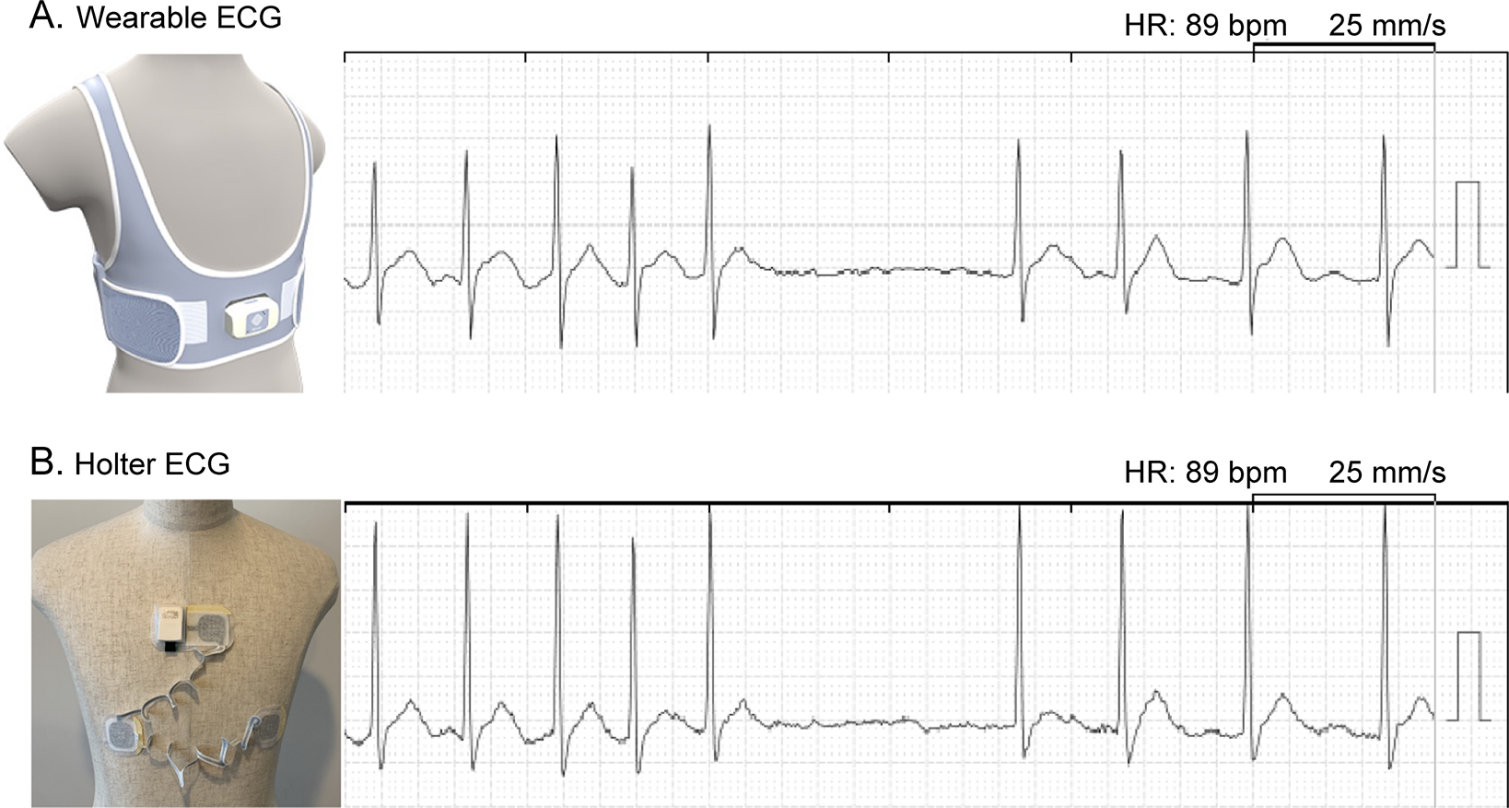
Simultaneous tracing from wearable ECG (A) and Holter ECG (B). Electrodes were positioned to compose a bipolar lead CC5 in both ECG devices. ECG, electrocardiogram; HR, heart rate.

A total of 18 patients with AF were included in this study ( Table 1). The majority of patients were men with non-paroxysmal AF. Two-thirds of AF ablation cases were completed within the first session. The most frequent CHADS _2_ score was 1. The CHADS2 score estimates stroke-risk in AF, which includes chronic heart failure, hypertension, diabetes, and age ≥75 years for one point each, and history of stroke or transient ischemic attack as two points. All patients completed the three-hour ambulatory ECG recording, which was simultaneously obtained from the wearable and Holter ECG. None of the patients demonstrated skin issues associated with the electrodes. Representative examples of simultaneous ECG are shown in Figure 2.

**Table 1.** Patient characteristics.

Demographics (N=18)	
**Age, years, mean ± SD**	66 ± 11
**Male, n (%)**	14 (78)
**BMI, kg/m** ^**2**^ **, mean ± SD**	23.8 ± 1.9
**Types of AF, n (%)**	
Paroxysmal AF	8 (44)
Persistent AF	8 (44)
Long-standing persistent AF	2 (11)
**Sessions of AF ablation, n (%)**	
First session	12 (67)
Second session	5 (28)
Third session	1 (5)
**CHADS**_**2**_ **score, n (%)**	
0	2 (11)
1	11 (61)
2	4 (22)
3	1 (6)

AF, atrial fibrillation; BMI, body mass index; CHADS _2_, Congestive heart failure, Hypertension, Age ≥75, diabetes mellitus, previous stroke/transient ischemic attack; SD, standard deviation.

**Figure 3.**
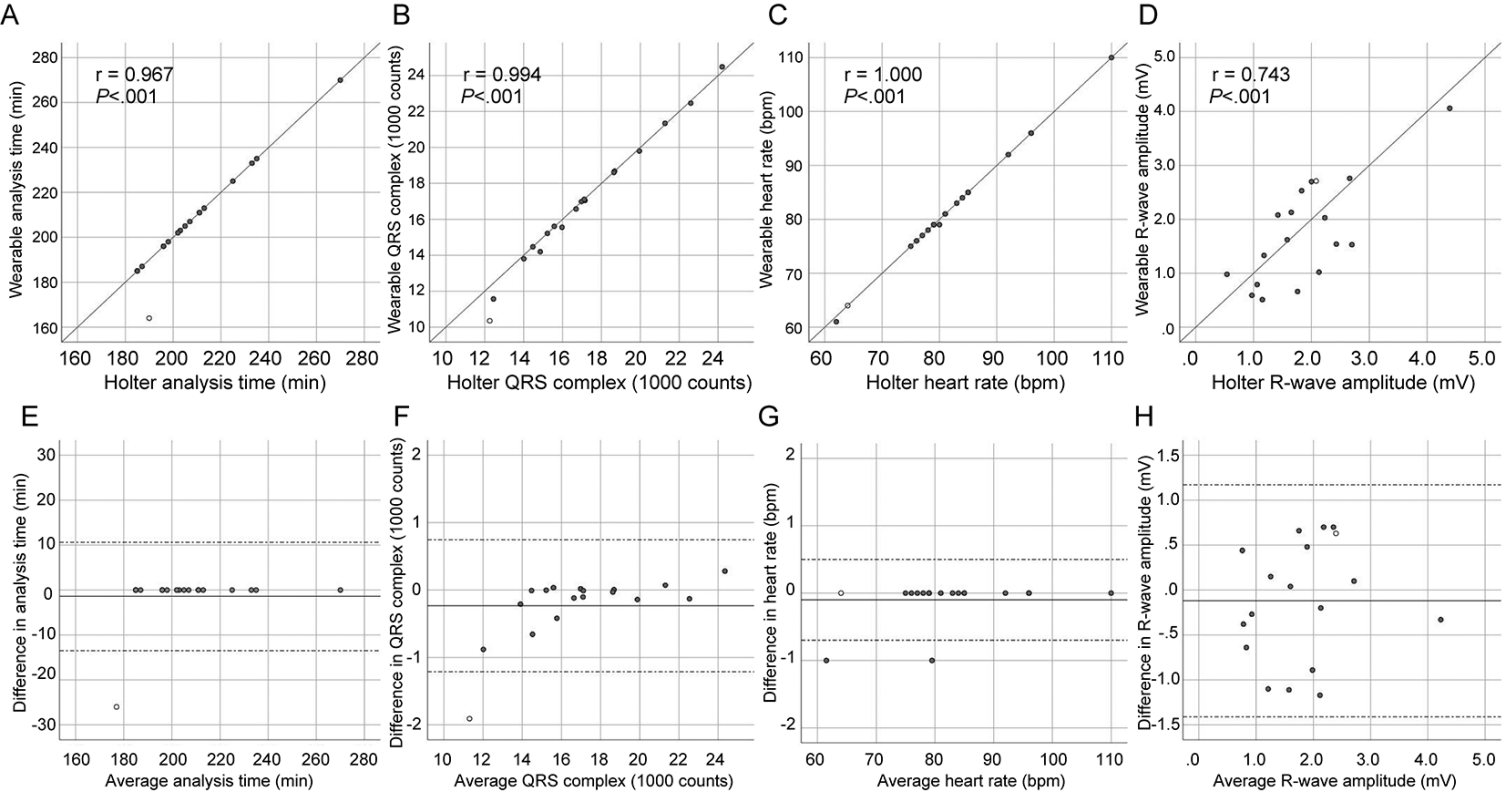
Correlation and Bland-Altman analysis between wearable and Holter ECGs. The scatterplot diagrams (A–D) illustrate correlation analysis with Pearson’s coefficient (r) and *P* values. The Bland-Altman plots (E–H) show a bias (solid line) with 95% limits of agreement (dotted line). Each dot represents paired values derived from all patients. An open circle indicates a patient with skin-electrode contact failure. ECG, electrocardiogram.

There was no difference in analysis time, the number of QRS complexes, heart rate, and R-wave amplitude between the devices ( Table 2), and almost complete correlation was demonstrated in those parameters ( Figure 3A–C), with the exception of R-wave amplitude ( Figure 3D). The Bland-Altman analysis revealed a small bias with narrow 95% limits of agreement in analysis time, the number of total QRS complexes, and heart rate between devices ( Figure 3E–G). R-wave amplitude, however, demonstrated poor agreement ( Figure 3H).

**Table 2.** Simultaneous analysis of ECG recordings.

Variables (N=18)	Wearable ECG	Holter ECG	*P*-value
**Parametric analysis**			
Total analysis time, min	207 ± 24	208 ± 21	.33
Total heart beats (per three hours)	16883 ± 3623	17116 ± 3298	.06
Average heart rate, bpm	82.2 ± 11.4	82.3 ± 11.3	.16
R-wave amplitude, mV	1.75 ± 0.96	1.88 ± 0.87	.44
**Non-parametric analysis**			
APC count (per three hours)	39 (6–83)	38 (5–83)	.54
APC burden, %	0.21 (0.03–0.48)	0.22 (0.03–0.48)	.27
VPC count (per three hours)	2 (0–33)	2 (0–34)	.72
VPC burden, %	0.01 (0.00–0.20)	0.01 (0.00–0.20)	.54
Noise count (per three hours)	113 (25–166)	9 (3–56)	.03
Noise burden, %	0.62 (0.15–1.11)	0.06 (0.02–0.34)	.02

APC, atrial premature complex; ECG, electrocardiogram; VPC, ventricular premature complex. Variables are shown as mean ± standard deviation or median (interquartile range).

The number of APCs and VPCs did not differ between the two devices ( Table 2). A very strong correlation in the number of APCs (ρ = 0.98; *P* < .001) and VPCs (ρ = 0.87; *P* < .001) was also demonstrated. The Passing-Bablok analysis demonstrated the absence of systemic and proportional bias in the number of APCs and VPCs ( Table 3). Noise signals, however, were more frequent in the wearable ECG than in the Holter ECG ( Table 2). There was no correlation between the two devices with regard to noise count (ρ = –0.16; *P* = .54). The agreement in noise count was not demonstrated ( Table 3). Furthermore, a difference in noise count was negatively correlated with the difference in R-wave amplitude (ρ = –0.52; *P* = .03).

**Table 3.** Passing-Bablock analysis between wearable ECG and Holter ECG.

Variables	Intercept (95% CI)	Slope (95% CI)
APC count (per three hours)	0.00 (–0.65–1.60)	1.00 (0.96–1.02)
VPC count (per three hours)	0.00 (–0.03–0.00)	1.00 (0.87–1.06)
Noise count (per three hours)	–295 (not calculated)	48.3 (not calculated)

APC, atrial premature complex; CI, confidence interval; ECG, electrocardiogram; VPC, ventricular premature complex.

An episode of skin contact failure of the textile electrode occurred in the fifth case (open circle in Figure 3). Accordingly, the wearable ECG demonstrated a shorter analysis time (164 vs. 190 min, Figure 3A, E) and a smaller number of QRS complexes (10,352 vs. 12,259 counts, Figure 3B, F) than the Holter ECG in this case. The patient was a non-obese (body mass index, 23.8 kg/m ^2^) 72-year-old man who underwent a second ablation for paroxysmal AF with a CHADS _2_ score of 1 for comorbid hypertension. A Velcro adjuster was then applied to the wearable ECG for all cases thereafter. This action prevented skin-electrode contact failure in the remaining 13 cases.

**Figure 4.**
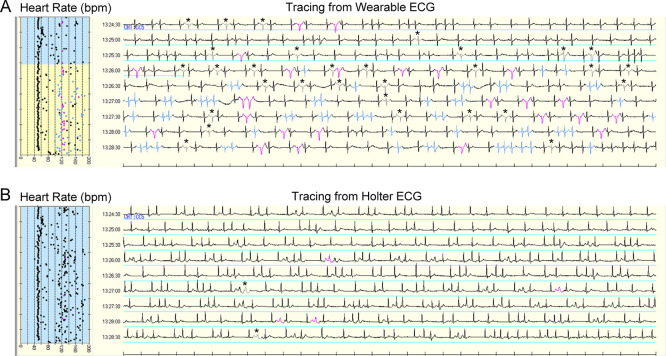
Simultaneous tracings of automatic interpretation from wearable ECG (A) and Holter ECG (B). Time in atrial fibrillation was indicated by the blue line. Most noise signals on wearable ECG were labeled as normal QRS complexes on Holter ECG. The gray signal with asterisk indicates noise; black signal, normal QRS complex; blue signal, atrial premature complex; pink signal, ventricular premature complex. ECG, electrocardiogram.

AF was detected in three patients. Two of these patients had a continuous AF episode. AF duration was completely equal between the two devices in both patients (196 and 225 min, respectively). In the remaining patient, the Holter ECG demonstrated seven AF episodes (99 min in total), whereas the wearable ECG detected nine AF episodes (96 min in total). AF episodes were fragmented by increased noise signals on the wearable ECG as compared to the Holter ECG (888 vs. 515 counts). Most of the increased noise signals on the wearable ECG were counted as normal QRS complexes on the Holter ECG ( Figure 4).

## Discussion

This pilot study evaluated whether wearable ECG using the hitoe ^®^ electrode would demonstrate simultaneous consistency with Holter ECG in a typical clinical setting. Ambulatory ECG monitoring after AF ablation was found to have generally consistent findings between the two devices ( Figure 3), although a slight discrepancy was found as follows. The wearable ECG demonstrated a 0.6% increase in noise signals on counting, which was related to a negative difference in R-wave amplitude as compared to the Holter ECG. The increased amount of noise signal caused a total 3-min (0.6%) interruption in recorded AF episodes. This is the first study demonstrating that the wearable ECG using the hitoe ^®^ electrode is a promising medical device for ambulatory AF monitoring without remarkable discrepancy against Holter ECG.

### Simultaneous consistency in clinical setting

This study evaluated the use of dry textile electrodes (hitoe ^®^) in ambulatory ECG monitoring for the first time in patients with AF. Wearable ECG devices are usually evaluated in healthy subjects for a very short monitoring period. ^14^^,^^15^ Use of a hitoe ^®^ textile ECG was previously reported to demonstrate consistency with Holter ECG in healthy volunteers for 3.5 min of sequential comparison. ^9^ Our study extended the feasibility of this ambulatory ECG monitoring period with hitoe ^®^ for up to three hours and expanded it into clinical settings, showing simultaneous consistency with Holter ECG ( Table 2, Figure 3). These results of inpatient ambulatory ECG monitoring after AF ablation warrant further study for outpatient applications.

### Noise signal and R-wave amplitude

Our simultaneous comparison revealed a slight increase in noise signals from hitoe ^®^ as compared to Holter ECG ( Table 2). Wearable ECG sensors were regarded previously as having 5–10% higher noise signals than Holter ECG during ambulatory monitoring. ^14^ Functional twisting movements were also reported to introduce up to 35% higher noise signals from a shirt-type ECG sensor with hitoe ^®^ as compared to Holter ECG. ^9^ Recently, a bra-type ECG sensor was reported to have better skin contact and signal quality versus a shirt-type sensor. ^15^ The bra-type ECG sensor using hitoe ^®^ equipped with a Velcro adjuster ( Figure 2) demonstrated only 0.6% higher noise signals against Holter ECG when measuring around 17 thousand beats during a three-hour simultaneous ambulatory monitoring period.

Bra-type equipment is widely used to improve ECG acquisition through textile electrodes because the electrodes are highly sensitive to motion artifacts. ^16^ Similar to our results, the R-wave amplitude has been reported to decrease with textile electrodes. ^16^^,^^17^ Better skin-electrode contact can result in a smaller R-wave amplitude; however, that change has been reported to be small. ^18^ Furthermore, ECG signals become clear with better skin-electrode contact. ^19^ Therefore, the skin-electrode contact should be maintained as much as possible. The potential risk of noise signal count increase owing to skin contact failure when using textile electrodes can be reduced by using a bra-type ECG with a Velcro adjuster. In contrast to the bra-type equipment, a shirt-type equipment covers the female breast tissue. The shirt type-equipment with hitoe ^®^ electrode was reported to demonstrate poor recorded data in few females during a marathon in a previous study. ^20^ The merit of bra-type equipment uncovering the breast tissue warrants further investigation to test a comparable ambulatory recording between female and male using hitoe ^®^ electrode.

An automatic analyzer (Kenz Cardy Analyzer 05 ^®^, SUZUKEN Co., Ltd.) revealed that increased noise signal counts were associated with decreased R-wave amplitude (ρ = –0.52; *P* = .03). Although hitoe ^®^ is both hydrophilic and flexible to enhance adequate skin contact, ^8^ textile electrodes remain vulnerable to motion artifacts that subsequently interfere with R wave detection. ^21^ Ousaka et al. reported that ECG acquisition by hitoe ^®^ electrode was better in mid-phase of running than in the early phase. ^20^ They speculated that a sweat production by running diminished a friction between the hydrophilic electrode and skin. Accordingly, the textile electrode was wetted with 30% glycerol aqueous solution before use to improve ECG acquisition. In addition, an amplitude of R wave against noise signal from hitoe ^®^ was reported to decrease during movement. ^9^ Despite the absence of a significant difference in the R-wave amplitude ( Table 2), a signal intensity that can fluctuate during ambulatory monitoring remains an issue of hitoe ^®^ ( Figure 3H).

### Detection of AF episode

Our simultaneous comparison revealed a fragmented interpretation of AF episodes in one patient ( Figure 4). On the wearable ECG, some R waves during AF episodes were interpreted as noise signals, which were excluded from the AF analysis. As discussed above, the under-detection of R waves resulted in increased noise signals in wearable ECG versus Holter ECG during ambulatory monitoring. ^22^ The increased noise signals fragmented AF episodes, which appeared continuous on the Holter ECG. However, the discrepancy in AF duration between the two devices was only 0.6% (3/520 min), owing to the hydrophilic and flexible nature of hitoe ^®^ and the bra-type ECG design with Velcro adjuster for skin-electrode contact. The 0.6% discrepancy in AF duration is clinically negligible for AF detection in long-term monitoring because the longer monitoring period will more than compensate for this slight discrepancy. Consequently, an ECG system using the hitoe ^®^ electrode seems quite amenable to long-term AF monitoring in clinical settings.

### Study limitations

One limitation of this pilot study was the small sample size. However, this is the first patient study demonstrating AF detection with the hitoe ^®^ electrode. In addition, this study included more participants (n=18) than the minimum sample size to detect a correlation coefficient (≥0.70) differing from zero (power 0.8; alpha 0.05) was 13 for Pearson’s and 15 for Spearman’s analysis. ^13^ Although the monitoring duration was limited to three hours, the continuous recording of ambulatory ECG for both devices was compared in a simultaneous fashion. Another limitation of this study was that it entailed an in-hospital ECG recording on the day after AF ablation. Although there was no restriction on physical activity, in-hospital ECG monitoring at this early time point might have reduced patient activity level and subsequently reinforced our highly consistent results. These issues should be addressed in future clinical trials.

## Conclusions

Monitoring of patients using a wearable ECG device that utilizes a medical-grade dry textile electrode (hitoe ^®^) provided simultaneous consistency with Holter ECG after AF ablation. The wearable ECG demonstrated a slight increase in noise signal episodes with associated deterioration in R-wave amplitude. This increased noise signal count caused a negligible interruption in a continuous AF episode in a patient with intermittent AF. Long-term monitoring of AF with wearable ECG warrants further clinical investigation.

## Data availability

The datasets generated and/or analyzed during the current study are not publicly available due to the limited permission from the participants but are available from the corresponding author if they have ethical approval for using the data according to the Japanese Ethical Guidelines for Medical and Health Research Involving Human Subjects. The ethical committee’s name and the approval number of the study are also required.

## Consent

Written informed consent for publication of the participants’ details and their images was obtained from the participants.
