# Supplementary material for: Dry textile electrode for ambulatory monitoring after catheter ablation of atrial fibrillation: A pilot study of simultaneous comparison to the Holter electrocardiogram
Source: F1000Res. 2022 Jun 30;11:97. Originally published 2022 Jan 26. [Version 2] doi: 10.12688/f1000research.75712.2 (PMC9263574; doi:10.12688/f1000research.75712.2)
Supplement: Peer review history

# Peer review: Reviewer response for version 2

**DOI:** 10.5256/f1000research.133916.r142812

**Published:** 2022-07-07

**Authors:** Katya Arquilla

**Affiliations:** 1 Department of Aeronautics and Astronautics, Massachusetts Institute of Technology, Cambridge, MA, USA

Thank you for addressing my recommendations in this updated version.

Is the work clearly and accurately presented and does it cite the current literature?

Yes

If applicable, is the statistical analysis and its interpretation appropriate?

Yes

Are all the source data underlying the results available to ensure full reproducibility?

No

Is the study design appropriate and is the work technically sound?

Yes

Are the conclusions drawn adequately supported by the results?

Partly

Are sufficient details of methods and analysis provided to allow replication by others?

Yes

Reviewer Expertise:

Bioastronautics, wearable sensor systems

I confirm that I have read this submission and believe that I have an appropriate level of expertise to confirm that it is of an acceptable scientific standard.

# Peer review: Reviewer response for version 1

**DOI:** 10.5256/f1000research.79621.r121137

**Published:** 2022-03-30

**Authors:** Katya Arquilla

**Affiliations:** 1 Department of Aeronautics and Astronautics, Massachusetts Institute of Technology, Cambridge, MA, USA

This study compares a wearable ECG monitoring system with the gold-standard Holter monitor system in the detection of atrial fibrillation during a 3-4 minute monitoring session. The article is clear and the study is sound, but a few small changes would help readers understand the work and implications:

1. Abstract, Background: "Limited recording time" really means lack of comfort for long-duration recordings, right?  Should be clearer with that distinction, because existing systems are capable of longer recording times, they're just not ideal for comfort.  
2. Introduction, paragraph 2, line 4: Should be more specific here about what threshold is required to deem something "very noisy".
3. Methods, Recruitment: Should state here how many males/females were tested, in addition to where it is shown in the table.
4. Study design: It would be helpful to show an image of the simultaneous placement of the two systems in this section. 
5. Measurements, paragraph 2, line 1: What was the approach to artifact removal?
6. Statistical analysis, paragraph 1, line 1: What are the categorical variables here?  Can you include an example in this sentence of which metrics are treated in this way?
7. Results, paragraph 1, line 2: "CHADS2" is not defined anywhere, so the reader will not necessarily know what this metric means.
8. Results, paragraph 2: Was signal-to-noise ratio not included in this analysis?
9. Figure 2: Were the instances of skin-electrode contact failure quantified and compared between the systems? 
10. Discussion, paragraph 1, line 4-6: A signal-to-noise ratio measurement would be helpful here.
11. Discussion, paragraph 2, line 4: Is there a plan for testing at the 24-hour scale in addition to this 3-minute test? 
12. Discussion, paragraph 3, line 5-7: Until this point, it wasn't clear that the bra-type wearable system was developed by this group and not the company that produces the electrodes. It would be good to make this distinction earlier on.
13. Discussion, paragraph 4: Given the bra-type wearable, it's important to test this system on both male and female participants, due to the added challenges during motion created by the presence of breast tissue. Is there any plan to test the system with more participants that have breast tissue?
14. Discussion, paragraph 5, line 1: Include specifications of this analyzer.
15. Discussion, paragraph 5, line 2: Does this hydrophilic characteristic impact the signal over time? I imagine it would, given the changes in electrical conductivity caused by sweat. 
16. Discussion, paragraph 6, line 7-8: Would this discrepancy scale linearly or would it become more of a problem in longer-duration tests?
17. Discussion, paragraph 7: It's mentioned earlier in the paper that some sort of power analysis was conducted to justify the sample size. It would be helpful to include that here.

Is the work clearly and accurately presented and does it cite the current literature?

Yes

If applicable, is the statistical analysis and its interpretation appropriate?

Yes

Are all the source data underlying the results available to ensure full reproducibility?

No

Is the study design appropriate and is the work technically sound?

Yes

Are the conclusions drawn adequately supported by the results?

Partly

Are sufficient details of methods and analysis provided to allow replication by others?

Yes

Reviewer Expertise:

Bioastronautics, wearable sensor systems

I confirm that I have read this submission and believe that I have an appropriate level of expertise to confirm that it is of an acceptable scientific standard, however I have significant reservations, as outlined above.

# Peer review: Reviewer response for version 1

**DOI:** 10.5256/f1000research.79621.r121135

**Published:** 2022-02-23

**Authors:** Takanori Arimoto

**Affiliations:** 1 The Department of Cardiology, Pulmonology, and Nephrology, Yamagata University School of Medicine, Yamagata, Japan

Monitoring of atrial fibrillation (AF) is important for the management of AF after catheter ablation. Machino *et al*. demonstrated the clinical usefulness of novel wearable ECG with a medical-grade dry textile electrode. There was almost complete correlation in the number of QRS complexes and heart rate. Importantly, the frequency of atrial premature complex (APC) count, APC burden, and AF recorded on the wearable device was highly consistent with the result of Holter ECG. On the other hand, some QRS complexes on the Holter ECG were sometimes shown as different QRS morphologies on the wearable ECG, as shown in Figure 3. The result of out-hospital ECG during daily physical activity may increase the credibility of this study. Wearable ECG monitoring may become a one of promising device in the near future.

Is the work clearly and accurately presented and does it cite the current literature?

Yes

If applicable, is the statistical analysis and its interpretation appropriate?

Yes

Are all the source data underlying the results available to ensure full reproducibility?

Yes

Is the study design appropriate and is the work technically sound?

Yes

Are the conclusions drawn adequately supported by the results?

Yes

Are sufficient details of methods and analysis provided to allow replication by others?

Yes

Reviewer Expertise:

Clinical electrophysiology, catheter ablation, device implantation.

I confirm that I have read this submission and believe that I have an appropriate level of expertise to confirm that it is of an acceptable scientific standard.
